# Relationship between cardiometabolic alterations and frailty in Brazilian urban and rural older adults

**DOI:** 10.1007/s40200-026-02046-8

**Published:** 2026-08-10

**Authors:** Jérsica Martins Bittencourt, Sarah Aparecida Vieira Ribeiro, Débora Carvalho Ferreira, Andréia Queiroz Ribeiro

**Affiliations:** 1 Candidate in Nutrition Science, Department of Nutrition and Health, Center for Biological and Health Sciences II (CCB II), Federal University of Viçosa (UFV), University Campus, Viçosa, MG, CEP 36570-900 Brazil; 2 Department of Nutrition and Health, Federal University of Viçosa, Viçosa, Minas Gerais Brazil; 3 Department of Medicine and Nursing, Federal University of Viçosa, Viçosa, Minas Gerais Brazil

**Keywords:** Abdominal obesity, Hypertension, Diabetes mellitus, Cardiovascular diseases, Frailty, Older adults

## Abstract

**Background:**

Frailty is a major public health concern among older adults due to its association with disability, hospitalization, and mortality. Cardiometabolic alterations are prevalent in this population and may be associated with frailty. However, evidence remains limited, particularly across different residential contexts.

**Objective:**

Investigate the association between cardiometabolic alterations and pre-frailty and frailty among Brazilian older adults.

**Methods:**

This cross-sectional study used data from the baseline survey (2015–2016) of the Brazilian Longitudinal Study of Aging (ELSI-Brazil), a nationally representative, population-based cohort study. The sample was selected using a multistage, stratified cluster sampling design. A total of 4,368 individuals aged 60 years were included. Frailty was assessed according to the Fried phenotype. Cardiometabolic alterations included diabetes mellitus, hypertension, high cholesterol, excess body weight, abdominal obesity, and cardiovascular risk. Multinomial logistic regression models were used to estimate crude and adjusted odds ratios (OR) and 95% confidence intervals (95% CI).

**Results:**

The prevalence of pre-frailty and frailty was 58.3% and 12.3%, respectively. Diabetes mellitus, hypertension, excess body weight, abdominal obesity, and the presence of three or more cardiometabolic alterations were independently associated with higher odds of pre-frailty and frailty after adjustment. A dose–response relationship was observed between the number of cardiometabolic alterations and frailty status.

**Conclusion:**

Cardiometabolic alterations are associated with pre-frailty and frailty among Brazilian older adults. The dose–response relationship between the number of alterations and frailty status highlights the relevance of accumulated cardiometabolic burden as a marker of vulnerability to frailty and supports integrated strategies for cardiometabolic risk prevention and management.

**Supplementary Information:**

The online version contains supplementary material available at 10.1007/s40200-026-02046-8.

## Introduction

Projections indicate a substantial global increase in the population of older adults in the coming decades, with a marked rise in the number of individuals aged 60 years or older by 2030 and a projected doubling of this population by 2050. Furthermore, the population aged 80 years and over is expected to grow even more rapidly during this period, potentially tripling [1].

In Brazil, the population aging process has occurred rapidly and markedly. According to data from the 2022 Demographic Census conducted by the Brazilian Institute of Geography and Statistics (IBGE), approximately 32 million Brazilians were aged 60 years or older, representing 15.8% of the country’s population. This group increased by more than 10 million individuals compared with the 2010 Census, highlighting a significant shift in the age structure of the Brazilian population and the progression of this demographic shift in the country [2].

Although this demographic transition reflects important advances in social and health development, it also poses significant challenges for health systems, which must adapt to emerging demands associated with population aging [1, 2]. In Brazil, the Unified Health System (Sistema Único de Saúde – SUS) is responsible for ensuring universal and comprehensive healthcare for the population and needs to reorganize its care models and strategies to meet the complex health needs of older adults and promote healthy aging [1, 3, 4].

In this context, frailty has emerged as an important public health concern, defined as a multidimensional syndrome characterized by progressive physiological decline and increased vulnerability to stressors and adverse events, including disability, hospitalization, and mortality [5–7]. Frailty prevalence is high worldwide and varies according to the population, conceptual model, and assessment instrument applied [7, 8]. In Brazil, a systematic review and meta-analysis of 28 studies, including 17,604 older adults, reported an overall frailty prevalence of 24% [9].

Several factors have been associated with the occurrence of frailty, including clinical conditions such as multimorbidity [7, 10]. Among these factors, non-communicable chronic diseases (NCDs), particularly cardiometabolic alterations such as diabetes, hypertension, and excess body weight, have received increasing attention due to their high prevalence among older adults and their potential contribution to the development and progression of frailty [10, 11].

Although growing interest has been directed toward the relationship between cardiometabolic alterations and frailty, important gaps remain in the literature [10, 12]. Some of them were identified in an international systematic review and are specially related to the combined assessment of multiple cardiometabolic alterations, the use of appropriate adjustments for confounding factors, and the consideration of diverse population contexts [10].

This last aspect is particularly relevant in Brazil, where older adults residing in rural areas face greater barriers to accessing and utilizing health services compared with those living in urban areas, partly due to the geographic dispersion of rural populations and greater socioeconomic vulnerability, which may compromise disease prevention, early diagnosis, and the appropriate management of health conditions [13, 14]. Despite these challenges, this population remains underrepresented in epidemiological research [13, 14].

Therefore, generating evidence on health conditions among older adults, particularly in rural populations, is essential to inform public policies and support the development of effective strategies for the prevention and management of cardiometabolic alterations and frailty, thereby contributing to healthy aging [1, 10].

This study aimed to investigate the association between cardiometabolic alterations and frailty among Brazilian older adults, considering place of residence.

## Methods

### Study design

This cross-sectional study used data from the baseline survey (2015–2016) of the Brazilian Longitudinal Study of Aging (ELSI-Brazil), a nationally representative, population-based cohort study of adults aged ≥ 50 years. To ensure national representativeness of urban and rural populations across municipalities of different population sizes, ELSI-Brazil employed a multistage, stratified cluster sampling design [15, 16].

The sampling process involved the sequential selection of municipalities, census tracts, and households, with municipalities stratified into four groups according to population size, resulting in the selection of 70 municipalities across the five major geographic regions of Brazil [15, 16].

Overall, ELSI-Brazil included 9,412 participants aged 50 years or older, of whom 1,140 (15.3%) resided in rural areas [15, 16]. Detailed information on the ELSI-Brazil study design and procedures has been published elsewhere [15, 16].

## Data collection

Data collection was conducted by trained and certified interviewers according to standardized ELSI-Brazil protocols at participants’ homes, including questionnaire administration, anthropometric measurements, and physical performance assessments [15]. Before data collection, pilot testing was undertaken to optimize the data collection instruments and procedures. During the data collection, quality assurance procedures were continuously applied to promote the accuracy, consistency, and reliability of the collected information. A detailed description of these standardized procedures is provided in the ELSI-Brazil operational manual (available at: http://elsi.cpqrr.fiocruz.br/) [16].

## Eligibility criteria

For the present study, the initial analytical sample comprised 5,432 participants aged 60 years or older from the baseline survey (2015–2016) of ELSI-Brazil. Eligible participants were non-institutionalized older adults residing in urban and rural areas of Brazilian municipalities.

Participants with insufficient information to classify the frailty phenotype were excluded from the analyses. However, participants unable to perform the gait speed or handgrip strength tests were classified according to the ELSI-Brazil operationalization of the Fried phenotype rather than being excluded [17, 18]. Participants with missing anthropometric measurements (weight, height or waist circumference) had the corresponding values recorded as missing, and no data imputation was performed. Cognitive impairment was not an exclusion criterion.

## Study variables

### Dependent variable

The dependent variable was frailty, assessed according to the phenotype proposed by Fried et al. (2001) [5]. Accordingly, specific variables were constructed for each of its components: unintentional weight loss, exhaustion, slow gait speed, muscle weakness, and low physical activity level, following methodologies previously applied in studies using ELSI-Brazil data [17, 18].

Unintentional weight loss was identified through self-report of weight loss in the previous three months without dietary restriction (yes/no). When present, the amount of weight lost was recorded, and unintentional weight loss was defined as a loss greater than 4.5 kg during this period [17, 18].

Exhaustion was assessed based on two questions from the Center for Epidemiological Studies–Depression Scale (CES-D), validated for the Brazilian population [19], addressing the frequency with which participants felt unable to carry out their usual activities or perceived that everything they did required a great effort during the previous week. Response options ranged from “rarely or none of the time” to “most of the time.” Exhaustion was defined as present when both questions were answered as “some of the time (3–4 days)” or “most of the time” [17–20].

Gait speed was measured as the time (in seconds) required to walk a distance of three meters, considering the lowest value from two attempts. Slow gait speed was defined as the highest quintile of time distribution, stratified by sex and height. Participants who were unable to perform the test were classified as slow gait speed [17, 18].

Handgrip strength was assessed using three measurements taken from the non-dominant hand with a hydraulic hand dynamometer (SH5001, SAEHAN Corporation). The highest value was used for analysis. Muscle weakness was defined as handgrip strength in the lowest quartile, adjusted for sex and body mass index (BMI) quartilhes. Participants who were bedridden or unable to perform the handgrip strength test were classified as having muscle weakness [17, 18].

Physical activity level was estimated in weekly metabolic equivalents and expressed in kilocalories using the short form of the International Physical Activity Questionnaire (IPAQ), validated for the Brazilian population [21]. This instrument assesses the duration and intensity (light, moderate, or vigorous) of activities performed during the previous week across different domains, including work, transportation, leisure, and household activities. Individuals in the lowest quintile of weekly energy expenditure, stratified by sex, were classified as having a low level of physical activity [21, 22].

Finally, participants were classified as “frail” if they presented three or more components, “pre-frail” if they presented one or two components, and “non-frail” if none of the phenotype components were present [5].

### Independent variables

Cardiometabolic alterations were considered as independent variables. These were assessed based on self-reported cardiometabolic diseases (diabetes mellitus, hypertension, and high cholesterol) and anthropometric measurements. Body Mass Index (BMI) was used to assess excess body weight, Waist Circumference (WC) as an indicator of abdominal obesity, and Waist-to-Height Ratio (WHtR) to estimate cardiovascular risk [23–26].

BMI was calculated as body weight (kg) divided by height squared (m²), and individuals with BMI ≥ 28.0 kg/m² were classified as having excess body weight [27]. WC was classified according to reference values for abdominal obesity: ≥ 94 cm for men and ≥ 80 cm for women [28]. WHtR was calculated as waist circumference (cm) divided by height (cm), adopting a cutoff point of ≥ 0.50 to indicate increased cardiovascular risk [29].

Additionally, a variable representing the number of cardiometabolic alterations was created and subsequently dichotomized based on its median.

### Potential confounding variables

Demographic and socioeconomic variables were analyzed, including age in years at the time of the interview (categorized into age groups: 60–69, 70–79, and ≥ 80 years), sex (male and female), place of residence (urban and rural), self-reported race/skin color (white and other [Black, Brown, Asian, and Indigenous]), education level and to per capita household income. Education was assessed through the question “What is the highest grade of school you have completed?” and subsequently categorized as incomplete primary education and complete primary education or higher. Per capita household income was obtained by self-report and calculated as the total monthly income of all household members divided by the total number of household residents. Subsequently, it was dichotomized at the sample median.

Lifestyle-related variables were also assessed, specifically, food consumption, smoking, and alcohol consumption, as potential confounding factors [30]. Regarding food consumption, the consumption of fruits and vegetables was analyzed, as the World Health Organization recommends a minimum intake of 400 g per day of these foods approximately equivalent to five daily servings as a strategy to reduce the risk of non-communicable chronic diseases [31–33].

Based on these recommendations, a variable for regular consumption of fruits and vegetables was created and categorized as “yes” or “no.” Adequate consumption (“yes”) was defined when the individual reported consuming fruits three or more times per day and vegetables two or more times per day, according to responses to the questions “In general, how many times per day do you usually eat fruit?” and “In general, how many times per day do you usually eat vegetables?”, respectively. In addition, this consumption pattern had to be reported on at least five days per week.

Smoking (current or past) and alcohol consumption were also assessed using the questions: “Do you currently smoke?” and “Have you smoked in the past?” (yes/no), as well as “How often do you usually consume alcoholic beverages?”, with response options categorized as yes (less than once a month and once or more per month) and no (never).

Finally, polypharmacy was assessed using the question: “How many regularly or continuously prescribed medicines have you used in the last two weeks?” Polypharmacy was defined as the concurrent use of five or more prescribed medicines (≥ 5) [34].

### Statistical analysis

The distribution of sociodemographic characteristics, lifestyle variables, and cardiometabolic alterations was estimated according to frailty prevalence, as well as according to cardiometabolic alterations. Comparisons between proportions were performed using the Pearson chi-square test with Rao–Scott correction to account for the complex sampling design.

To assess the association between cardiometabolic alterations and frailty status, including pre-frailty and frailty categories, multinomial logistic regression analyses were performed. Crude and adjusted models were fitted separately for each cardiometabolic alteration, with frailty status categorized as robust, pre-frail, and frail, using robust participants as the reference category. Adjustment variables were selected a priori based on the literature and according to statistically significant differences observed in bivariate analyses. The models estimated Odds Ratio (OR) and their corresponding 95% confidence intervals (95% CI) for pre-frailty and frailty compared with the robust category.

All analyses were performed using R software version 4.6.0, with the survey and svyVGAM packages, accounting for the complex sampling design and individual sampling weights of the ELSI-Brazil study. Analyses incorporated sampling weights, primary sampling units, and strata to obtain unbiased population-level estimates.

A significance level of 5% (α = 0.05) was adopted for all hypothesis tests.

### Ethical considerations

ELSI-Brazil fully complies with the ethical standards established by the Research Ethics Committee of the René Rachou Institute, Oswaldo Cruz Foundation (CAAE No. 34649814.30000.5091). All participants included in the study provided written informed consent prior to participation [15].

## Results

A total of 4,368 participants from the first wave of ELSI-Brazil (2015–2016) with complete data on the components of the frailty phenotype were included.

The predominant age group was 60–69 years (58.9%). More than half of the participants were female (52.8%) and self-identified as non-white (Black, Brown, Asian, or Indigenous) (54.5%). Most participants had low educational attainment (incomplete primary education) (71.8%), lived in urban areas (83.3%), reported inadequate consumption of fruits and vegetables (95.3%), and did not report alcohol consumption (74.6%). More than half reported current or past smoking (54.0%), and most participants did not report polypharmacy (82.3%). The median household income per capita was R$821.66, with 51.5% of participants classified in the higher-income group and 48.5% in the lower-income group (Table 1).

A higher proportion of women, individuals with lower educational attainment, and participants with lower household income was observed in rural areas compared to urban areas (47.3% vs. 53.9%, *p* = 0.003; 92.2% vs. 67.8%, *p* < 0.001; and 89.5% vs. 77.5%, *p* < 0.001, respectively). (eTable 1).

Most participants were classified as pre-frail (58.3%), followed by non-frail (29.4%) and frail (12.3%). Significant differences in frailty status were observed according to sociodemographic characteristics and lifestyle factors. Among frail individuals, there was a higher proportion of older adults aged ≥ 80 years (26.2% vs. 11.8% among pre-frail and 4.2% among non-frail), females (59.7% vs. 56.7% and 42.4%), and those with low educational attainment (85.0% vs. 73.5% and 62.8%), those with lower household income (56.6% vs. 49.8% and 42.3%), and those reporting polypharmacy (29.3% vs. 18.6% and 11.0%), respectively.

In addition, a lower frequency of current or past smoking was observed among frail individuals (51.4% vs. 53.1% and 57.8%), as well as lower alcohol consumption (12.9% vs. 22.8% and 35.8%), compared to pre-frail and non-frail individuals, respectively. No significant differences were found regarding place of residence or inadequate consumption of fruits and vegetables (Table 1).

**Table 1:** Demographic, socioeconomic, lifestyle, and polypharmacy characteristics according to frailty status among Brazilian older adults. ELSI-Brazil, 2015–2016

Characteristics	*n*	Total (%)	Non-frail (%)	Pre-frail (%)	Frail (%)	*p*-value*
Age group (years)	4,368					< 0.001
60–69		58.9	70.9	57.6	36.3	
70–79		29.8	24.9	30.6	37.5	
≥ 80		11.3	4.2	11.8	26.2	
Sex	4,368					< 0.001
Female		52.8	42.4	56.7	59.7	
Male		47.2	57.6	43.3	40.3	
Self-reported race/skin color	4,203					0.894
White		45.5	45.9	44.1	45.6	
Other**		54.5	54.1	55.9	54.4	
Education	4,340					< 0.001
Up to incomplete primary education		71.8	62.8	73.5	85.0	
Complete primary education or more		28.2	37.2	26.5	15.0	
Household income per capita	4,368					< 0.001
< R$ 821,66		48.5	42.3	49.8	56.6	
≥ R$ 821,66		51.5	57.7	50.2	43.4	
Place of residence	4,368					0.946
Urban		83.3	83.3	83.8	83.9	
Rural		16.7	16.7	16.2	16.1	
Low consumption of fruits and vegetables	4,312					0.661
Yes		95.3	95.1	95.5	94.4	
No		4.7	4.9	4.5	5.6	
Current or former smoking	4,365					0.136
Yes		54.0	57.8	53.1	51.4	
No		46.0	42.2	46.9	48.6	
Alcohol consumption	4,366					< 0.001
Yes		25.4	35.8	22.8	12.9	
No		74.6	64.2	77.2	87.1	
Polypharmacy	4,350					< 0.001
Yes		17.7	11.0	18.6	29.3	
No		82.3	89.0	81.4	70.7	

Frailty status was defined according to the phenotype proposed by Fried et al. Abbreviations: n, sample size. Footnotes: a Percentages refer to weighted proportions within each frailty category; b Incomplete frailty phenotype data were excluded, and sample size may vary across variables due to missing data; c Frailty classification: non-frail (0 criteria), pre-frail (1–2 criteria), frail (≥ 3 criteria). *p-values were calculated using Pearson’s chi-square test with Rao–Scott correction. **Individuals self-identifying as Black, Asian, Brown (mixed race), or Indigenous

In eTable 2, significant differences were observed in the prevalence of cardiometabolic alterations according to sociodemographic and lifestyle characteristics. The prevalence of diabetes mellitus was higher among urban residents compared to rural residents (19.8% vs. 11.4%), among non-smokers compared to smokers (19.2% vs. 14.2%), and among those who did not consume alcohol compared to those who did (19.5% vs. 15.5%) and higher among individuals with polypharmacy compared with those without polypharmacy (35.8% vs. 14.6%).

Hypertension was more frequent among individuals aged 70–79 years compared to those aged 60–69 years and those aged 80 years or older (65.2% vs. 58.6% and 61.1%, respectively), among females compared to males (64.8% vs. 56.4%), among individuals with complete primary education or higher compared to those with lower educational attainment (62.8% vs. 55.7%), among non-smokers compared to smokers (62.7% vs. 49.7%), among those who did not consume alcohol compared to those who did (63.1% vs. 54.2%) and among individuals with polypharmacy compared with those without polypharmacy (81.5% vs. 56.4%).

The prevalence of high cholesterol was higher among individuals aged 60–69 years compared to older age groups (34.9% vs. 30.3% among those aged 70–79 years and 23.4% among those aged ≥ 80 years), among females compared to males (40.3% vs. 23.1%), among non-white individuals compared to white individuals (34.3% vs. 30.3%), among urban residents compared to rural residents (33.0% vs. 28.0%), among non-smokers compared to smokers (33.4% vs. 24.8%), among those who did not consume alcohol compared to those who did (34.0% vs. 26.9%) and among individuals with polypharmacy compared with those without polypharmacy (42.2% vs. 30.0%).

Excess body weight was more prevalent among females compared with males (50.0% vs. 35.7%), among individuals with a household income per capita ≥ R$ 821.66 compared with those with lower income (45.7% vs. 40.6%), among urban residents compared with rural residents (45.2% vs. 33.6%), among those with adequate consumption of fruits and vegetables compared with those with low consumption (56.6% vs. 42.6%), among non-smokers compared with smokers (46.3% vs. 25.1%), among those who did not consume alcohol compared with those who did (44.7% vs. 39.3%), and among individuals with polypharmacy compared with those without polypharmacy (56.7% vs. 40.3%).

Abdominal obesity was more frequent among females compared to males (83.5% vs. 56.6%), among urban residents compared to rural residents (72.5% vs. 62.3%), among non-smokers compared to smokers (74.4% vs. 49.0%), among those who did not consume alcohol compared to those who did (72.5% vs. 65.8%), among individuals with a household income per capita ≥ R$ 821.66 compared with those with lower income (74.0% vs. 67.6%) and among individuals with polypharmacy compared with those without polypharmacy (82.7% vs. 68.3%).

Cardiovascular risk was more prevalent among females compared to males (57.1% vs. 40.1%), among individuals with complete primary education or higher compared to those with lower educational attainment (50.4% vs. 45.6%), among individuals with a household income per capita ≥ R$ 821.66 compared with those with lower income (39.4% vs. 37.8%) among urban residents compared to rural residents (50.7% vs. 40.7%), among non-smokers compared to smokers (52.3% vs. 29.4%), among those who did not consume alcohol compared to those who did (51.5% vs. 41.9%), among individuals with inadequate consumption of fruits and vegetables compared to those with adequate consumption (56.6% vs. 43.4%) and among individuals with polypharmacy compared with those without polypharmacy (62.3% vs. 17.9%).

According to Table 2, cardiometabolic alterations were highly prevalent among older adults in Brazil, particularly cardiovascular risk (88,5%) and abdominal obesity (71,0%), hypertension (60.9%), and excess body weight (43.2%). Additionally, nearly two-thirds of participants presented three or more cardiometabolic alterations.

The occurrence of frailty was higher among individuals with diabetes mellitus (16.7%) and hypertension (13.9%) compared to those without these conditions (11.3% and 9.7%, respectively; *p* < 0.001). A higher prevalence of frailty was also observed among older adults with excess body weight (13.3%), abdominal obesity (12.8%), and increased cardiovascular risk (12.5%) compared to those without these conditions (11.4%, 11.0%, and 10.0%, respectively; *p* < 0.001).

A similar pattern was observed for pre-frailty, with consistently higher prevalence among individuals with cardiometabolic alterations, particularly those with diabetes (60.8%), hypertension (60.2%) and abdominal obesity (61.2%).

Among frail individuals, a higher proportion of participants presented a greater number of cardiometabolic alterations. Specifically, 57.7% of frail individuals had four or more cardiometabolic alterations, compared with 47.0% of pre-frail and 32.7% of robust individuals. Furthermore, dose–response relationship was observed between the accumulation of cardiometabolic alterations and frailty status (*p* < 0.001) (Table 2).

In a complementary analysis, the prevalence of pre-frailty increased progressively from 50.9% among individuals without cardiometabolic alterations to 70.5% among those with six alterations. Similarly, the prevalence of frailty increased from 5.7% to 14.9%, respectively.

**Table 2:** Prevalence of frailty according to cardiometabolic alterations among Brazilian older adults. ELSI-Brazil, 2015–2016

Cardiometabolic alterations	*n*	Brazil (%)	Non-frail (%)	Pre-frail (%)	Frail (%)	*p*-value*
Diabetes mellitus	4,348					< 0.001
Yes		18.4	22.5	60.8	16.7	
No		81.6	31.0	57.7	11.3	
Hypertension	4,360					< 0.001
Yes		60.9	25.9	60.2	13.9	
No		39.1	34.9	55.4	9.7	
High cholesterol	4,333					0.222
Yes		32.2	27.4	60.8	11.8	
No		67.8	30.5	57.1	12.4	
Excess body weight	4,368					< 0.001
Yes		43.2	35.5	65.2	13.3	
No		56.7	21.5	53.1	11.4	
Abdominal obesity	4,368					< 0.001
Yes		71.0	26.0	61.2	12.8	
No		29.0	37.7	51.3	11.0	
Cardiovascular risk	4,362					< 0.001
Yes		88.6	28.3	59.2	12.5	
No		11.4	38.5	51.5	10.0	
Number of cardiometabolic alterations	4,368					< 0.001
0		6.6	9.8	5.7	3.1	
1		12.3	15.7	10.5	12.7	
2		16.4	20.3	15.0	14.1	
3		22.2	22.9	21.8	22.5	
4		25.0	19.3	27.6	25.8	
5		14.8	10.6	16.0	18.5	
6		2.8	1.4	3.4	3.4	

Frailty status was defined according to the phenotype proposed by Fried et al. Abbreviation: n, sample size. Footnotes: a Incomplete frailty phenotype data were excluded, and sample size may vary across variables due to missing data. *p-values were calculated using Pearson’s chi-square test with Rao–Scott correction

Table 3 shows the crude and adjusted odds ratios (OR) for the association between cardiometabolic alterations and pre-frailty and frailty. In the adjusted models, diabetes mellitus, hypertension, excess body weight, abdominal obesity, and the presence of three or more cardiometabolic alterations remained significantly associated with higher odds of both pre-frailty and frailty compared with robust participants. Cardiovascular risk remained significantly associated with pre-frailty but was no longer associated with frailty after adjustment. Elevated cholesterol was not significantly associated with pre-frailty or frailty after adjustment.

**Table 3:** Crude and adjusted associations between cardiometabolic alterations and pre-frailty and frailty among Brazilian older adults. ELSI-Brazil, 2015–2016

/Variables	Pre-frailty (Crude) OR (95% CI)	Pre-frailty (Adjusted†) OR (95% CI)	Frailty (Crude) OR (95% CI)	Frailty (Adjusted†) OR (95% CI)
Diabetes mellitus	1.45 (1.17–1.81)	1.32 (1.05–1.66)	2.05 (1.51–2.78)	1.64 (1.16–2.32)
Hypertension	1.46 (1.23–1.74)	1.28 (1.06–1.56)	1.94 (1.52–2.47)	1.56 (1.14–2.15)
High cholesterol	1.19 (0.97–1.45)	1.09 (0.88–1.36)	1.07 (0.79–1.44)	1.07 (0.77–1.48)
Excess body weight	2.04 (1.75–2.37)	1.99 (1.68–2.36)	1.93 (1.56–2.39)	1.98 (1.56–2.50)
Abdominal obesity	1.72 (1.46–2.03)	1.52 (1.25–1.85)	1.68 (1.28–2.21)	1.50 (1.04–2.17)
Cardiovascular risk	1.59 (1.29–1.96)	1.38 (1.13–1.70)	1.69 (1.14–2.50)	1.36 (0.86–2.14)
≥ 3 cardiometabolic alterations	1.86 (1.60 − 2.16)	1.65 (1.38 − 1.96)	2.03 (1.60 − 2.58)	1.78 (1.34 − 2.36)

OR Odds Ratios; *CI* confidence interval. Multinomial logistic regression models were performed separately for each exposure variable. †Adjusted for age, household income per capita, sex, self-reported race/skin color, education, place of residence, polypharmacy, low consumption of fruits and vegetables, smoking status, and alcohol consumption. Reference category: robust participants

## Discussion

The findings of this study demonstrated that diabetes mellitus, hypertension, excess body weight, abdominal obesity, and the presence of three or more cardiometabolic alterations were significantly associated with higher odds of both pre-frailty and frailty, even after adjustment for sociodemographic, socioeconomic, lifestyle, and polypharmacy factors. Cardiovascular risk was independently associated with pre-frailty but not with frailty after adjustment. These findings suggest that individual cardiometabolic alterations and the accumulation of multiple alterations are associated with an increased likelihood of pre-frailty and frailty among older adults.

An important finding of this study was the dose–response relationship between the number of cardiometabolic alterations and the prevalence of frailty. Although individual cardiometabolic conditions, were independently associated with pre-frailty and frailty, the progressive increase in frailty prevalence according to the accumulation of cardiometabolic alterations suggests an additional burden associated with multimorbidity. This pattern suggests that the accumulation of cardiometabolic alterations is associated with greater physiological vulnerability and a higher prevalence of frailty among older adults.

Studies investigating these relationships in representative samples are still limited, particularly those incorporating adjustments for sociodemographic factors, including place of residence and lifestyle behaviors [35]. Nevertheless, the findings observed in the present study are consistent with the international literature.

Jayanama et al. (2022), analyzing data from the NHANES and SHARE cohorts, demonstrated that overweight and obesity are associated with higher prevalence of frailty [36]. Similarly, a systematic review conducted by Shakya et al. (2024) found that abdominal obesity, hyperglycemia, and the presence of multiple cardiometabolic alterations increase the likelihood of frailty among older adults. In contrast, no significant association between high cholesterol and frailty was observed, which is consistent with the findings of the present study [35].

Although cardiovascular risk was no longer statistically significantly associated with frailty after adjustment, the point estimate remained above one, suggesting a possible positive association. This attenuation may reflect the multifactorial nature of frailty, in which the cumulative burden of chronic diseases and age-related physiological changes may reduce the independent contribution of cardiovascular risk alone [5, 18]. In contrast, the persistent association between cardiovascular risk and pre-frailty suggests that this stage may represent a critical window for implementing preventive strategies before the onset of frailty.

The lack of association between high cholesterol and frailty may be explained, at least in part, by the characteristics of the variable used in this study. High cholesterol was assessed by self-report, which depends on previous diagnosis and participants’ awareness of their condition. In addition, the use of lipid-lowering medications among older adults may attenuate the relationship between hypercholesterolemia and frailty by controlling serum lipid levels. Furthermore, the measure did not differentiate between lipid fractions (e.g., total cholesterol, LDL-C, HDL-C, or triglycerides) [37, 38].

The relationship between cardiometabolic alterations and frailty is recognized as bidirectional, with mutual influence across the life course [39]. Conditions such as type 2 diabetes mellitus and insulin resistance contribute to chronic hyperglycemia, low-grade systemic inflammation, and the loss of muscle mass and strength (sarcopenia) [39–42]. Hypertension is associated with vascular stiffness and reduced tissue perfusion, thereby accelerating functional decline [39], while obesity, particularly visceral obesity promotes chronic inflammation and muscle catabolism [39, 43].

Conversely, frailty may impair metabolic and cardiovascular control, increase vulnerability to adverse treatment effects, and intensify the risk of unfavorable clinical outcomes, including the onset or worsening of cardiometabolic alterations [32, 39, 44].

Furthermore, chronic low-grade systemic inflammation (inflammaging) contributes to anorexia, muscle catabolism, and reduced capacity to recover from stressors. This pro-inflammatory state promotes insulin resistance and muscle mass loss, leading to the development of frailty, while frailty itself may further amplify inflammatory responses, reinforcing this deleterious cycle [39, 42]. In parallel, metabolic diseases accelerate mitochondrial dysfunction, characterized by impaired mitochondrial biogenesis, increased production of reactive oxygen species, and energy deficits in skeletal muscle. These alterations limit exercise capacity, physiological resilience, and recovery from stress [39, 44].

Frailty was more prevalent among older adults aged 80 years or older, females, and individuals with lower educational attainment. Cardiometabolic alterations were also associated with sociodemographic characteristics. These findings reinforce that biological and social factors interact to increase vulnerability and should be jointly considered in strategies aimed at the prevention and care of older adults [18].

Additionally, descriptive analyses indicated a high prevalence of cardiometabolic alterations in both urban and rural areas, with a significantly greater magnitude in urban populations. Specifically, urban residence was associated with a higher prevalence of diabetes mellitus, excess body weight, abdominal obesity, and cardiovascular risk. These findings may be explained by contextual differences between urban and rural environments. In urban areas, the environment tends to be more obesogenic, characterized by more sedentary lifestyles, greater availability and consumption of ultra-processed foods, higher levels of stress, and increased exposure to environmental pollutants—factors that favor the development of cardiometabolic alterations [45, 46].

In contrast, although rural populations often face more limited access to healthcare services, this context may be partially offset by more physically active lifestyles and less industrialized dietary patterns, which may exert a protective effect and contribute to a lower magnitude of these alterations [13, 47, 48]. However, these findings should be interpreted with caution, as healthcare access disparities may contribute to underdiagnosis and underestimation of cardiometabolic conditions among rural residents [13, 14].

National and international studies corroborate these urban–rural differences. In Brazil, population-based analyses have reported a higher prevalence of general and abdominal obesity in urban areas compared to rural areas [47–49]. Internationally, a higher prevalence of overweight, obesity, and diabetes mellitus has also been observed in urban settings, even after adjustment for sociodemographic factors [49, 50].

An association was also observed between non-consumption of alcohol, non-smoking, and cardiometabolic alterations; however, this finding should be interpreted with caution. A similar unexpected pattern was observed for excess body weight, which was more prevalent among individuals without low consumption of fruits and vegetables. This finding may reflect reverse causality, as individuals with excess weight may have modified their dietary habits. In older populations, these behaviors tend to cluster among healthier individuals, reflecting a healthy selection effect, survival bias, and possible reverse causality. Therefore, the inverse associations observed likely do not represent causal protective effects and are limited by the lack of temporality inherent to the cross-sectional design [51].

Nevertheless, a high frequency of inadequate consumption of fruits and vegetables, current or past smoking, and alcohol consumption was observed, reinforcing the need for early prevention and intervention strategies that integrate the management of cardiometabolic alterations with the promotion of healthy lifestyle behaviors [1, 10, 52].

In this context, public policies that promote early detection, appropriate clinical follow-up, regular physical activity, healthy eating, and targeted actions for more vulnerable groups are essential to reduce the progression of frailty, minimize impacts on functionality, autonomy, and quality of life, and promote healthy aging, considering the specificities of both urban and rural contexts [1, 10].

Finally, this study has several important strengths, including the use of a nationally representative dataset of Brazilian older adults and the individual analysis of a set of cardiometabolic alterations selected based on scientific evidence. In addition, the anthropometric and clinical markers used are widely recognized for their relevance in assessing cardiometabolic alterations [23–26].

To our knowledge, this is the first Brazilian study to investigate the association between cardiometabolic alterations and frailty among older adults living in both urban and rural areas, using a nationally representative population-based sample and accounting for multiple potential confounding factors. Furthermore, our findings contribute to the international literature by providing evidence from a middle-income country with substantial urban–rural disparities, a context that remains underrepresented in studies investigating the relationship between cardiometabolic burden and frailty.

However, some limitations should be acknowledged. The cross-sectional design precludes establishing temporal directionality or causal relationships between cardiometabolic alterations and frailty status. In addition, the use of self-reported information for some health conditions may have introduced recall bias and potential misclassification, as awareness of previous diagnoses may vary among participants. This limitation may have attenuated some associations, particularly for high cholesterol, which may also be affected by pharmacological treatment and the lack of information on specific lipid fractions. Furthermore, the urban–rural classification may not fully capture the complexity and heterogeneity of living environments. Therefore, findings related to place of residence should be interpreted cautiously, as this variable may reflect a combination of individual and contextual characteristics that were not fully captured in this study.

The methodological strengths of ELSI-Brazil, including its population-based design, standardized data collection procedures, and national representativeness, strengthen the validity of these findings. Although the results can be generalized to Brazilian older adults living in different residential contexts, caution is warranted when extrapolating them to other age groups or populations with specific clinical characteristics.

## Conclusion

Using a nationally representative sample of Brazilian older adults living in urban and rural areas, this study demonstrated that diabetes mellitus, hypertension, excess body weight, abdominal obesity, and the accumulation of cardiometabolic alterations were independently associated with pre-frailty and frailty. The observed dose–response relationship between the number of cardiometabolic alterations and frailty status highlights the potential role of multimorbidity-related cardiometabolic burden.

These findings emphasize the importance of integrated approaches aimed at preventing, identifying, and controlling cardiometabolic alterations as potential strategies to reduce the occurrence and progression of frailty across different residential contexts.

## Supplementary Information

Below is the link to the electronic supplementary material.

Supplementary Material 1 (DOCX 17.8 KB)

Supplementary Material 2 (DOCX 18.5 KB)

## Data Availability

The data used in this study are available to researchers upon registration and approval through the ELSI-Brazil project portal.
