# Supplementary material for: Relationship between cardiometabolic alterations and frailty in Brazilian urban and rural older adults
Source: J Diabetes Metab Disord. 2026 Aug 10;25(2):230. doi: 10.1007/s40200-026-02046-8 (PMC13457821; doi:10.1007/s40200-026-02046-8)
Supplement: Supplementary file 1 [file 40200_2026_2046_MOESM1_ESM.docx]

**Supplementary Material**

**Journal of Diabetes & Metabolic Disorders**

**Relationship between cardiometabolic alterations and frailty in Brazilian urban and rural older adults**

Jérsica Martins Bittencourt¹; Sarah Aparecida Vieira Ribeiro^2^, Débora Carvalho Ferreira^3^; Andréia Queiroz Ribeiro³

¹ PhD Candidate in Nutrition Science, Department of Nutrition and Health, Federal University of Viçosa, Viçosa, Minas Gerais, Brazil.
Email: jersica.cunha@ufv.br | ORCID: 0000-0003-3821-8171

² Full Professor, Department of Nutrition and Health, Federal University of Viçosa, Viçosa, Minas Gerais, Brazil.
Email: sarah.vieira@ufv.br | ORCID: 0000-0002-0304-2711

³ Full Professor, Department of Medicine and Nursing, Federal University of Viçosa, Viçosa, Minas Gerais, Brazil.
Email: deboracarvalho@ufv.br | ORCID: 0000-0003-3348-3267

⁴ Full Professor, Department of Nutrition and Health, Federal University of Viçosa, Viçosa, Minas Gerais, Brazil.
Email: andreia.ribeiro@ufv.br | ORCID: 0000-0001-6546-1252

**Corresponding Author:** Jérsica Martins Bittencourt, PhD Candidate in Nutrition Science, Federal University of Viçosa (UFV), Department of Nutrition and Health, Center for Biological and Health Sciences II (CCB II), University Campus, Viçosa, MG, Brazil, CEP 36570-900; Phone: +55 (31) 3612-5186; Fax: +55 (31) 3612-5187; Email: jersica.cunha@ufv.br.

**eTable 1.** Demographic and socioeconomic characteristics of Brazilian older adults according to place of residence. ELSI-Brazil, 2015–2016.

| **Sociodemographic characteristics** | **n** | **Brazil (%)** | **Urban (%)** | **Rural (%)** | **p-value*** |
| --- | --- | --- | --- | --- | --- |
| **Age group (years)** | 4,368 |  |  |  |  |
| 60–74 |  | 58.9 | 25.7 | 40.2 | 0.655 |
| ≥75 |  | 41.1 | 74.3 | 59.8 |  |
| **Sex** | 4,368 |  |  |  |  |
| Female |  | 52.8 | 53.9 | 47.3 | 0.003 |
| Male |  | 47.2 | 46.1 | 52.7 |  |
| **Self-reported race/skin color** | 4,203 |  |  |  |  |
| White |  | 45.5 | 47.7 | 34.6 | 0.108 |
| Other** |  | 54.5 | 52.3 | 65.4 |  |
| **Education** | 4,340 |  |  |  |  |
| Less than complete primary education |  | 71.8 | 67.8 | 92.2 | <0.001 |
| Complete primary education or higher |  | 28.2 | 32.2 | 7.8 |  |
| **Household income per capita** | 4,368 |  |  |  |  |
| < R$ 821.66 |  | 48.5 | 77.5 | 89.5 | <0.001 |
| ≥ R$ 821.66 |  | 51.5 | 22.5 | 10.5 |  |

Note: *Pearson’s chi-square test with Rao–Scott correction.**Individuals self-identified as Black, Asian, Brown (mixed race), or Indigenous.
