# Supplementary material for: Relationship between cardiometabolic alterations and frailty in Brazilian urban and rural older adults
Source: J Diabetes Metab Disord. 2026 Aug 10;25(2):230. doi: 10.1007/s40200-026-02046-8 (PMC13457821; doi:10.1007/s40200-026-02046-8)
Supplement: Supplementary file 2 [file 40200_2026_2046_MOESM2_ESM.docx]

**Supplementary Material**

**Journal of Diabetes & Metabolic Disorders**

**Relationship between cardiometabolic alterations and frailty in Brazilian urban and rural older adults**

Jérsica Martins Bittencourt¹; Sarah Aparecida Vieira Ribeiro^2^, Débora Carvalho Ferreira^3^; Andréia Queiroz Ribeiro³

¹ PhD Candidate in Nutrition Science, Department of Nutrition and Health, Federal University of Viçosa, Viçosa, Minas Gerais, Brazil.
Email: jersica.cunha@ufv.br | ORCID: 0000-0003-3821-8171

² Full Professor, Department of Nutrition and Health, Federal University of Viçosa, Viçosa, Minas Gerais, Brazil.
Email: sarah.vieira@ufv.br | ORCID: 0000-0002-0304-2711

³ Full Professor, Department of Medicine and Nursing, Federal University of Viçosa, Viçosa, Minas Gerais, Brazil.
Email: deboracarvalho@ufv.br | ORCID: 0000-0003-3348-3267

⁴ Full Professor, Department of Nutrition and Health, Federal University of Viçosa, Viçosa, Minas Gerais, Brazil.
Email: andreia.ribeiro@ufv.br | ORCID: 0000-0001-6546-1252

**Corresponding Author:** Jérsica Martins Bittencourt, PhD Candidate in Nutrition Science, Federal University of Viçosa (UFV), Department of Nutrition and Health, Center for Biological and Health Sciences II (CCB II), University Campus, Viçosa, MG, Brazil, CEP 36570-900; Phone: +55 (31) 3612-5186; Fax: +55 (31) 3612-5187; Email: jersica.cunha@ufv.br.

**eTable 2.** Prevalence of cardiometabolic alterations according to sociodemographic and lifestyle characteristics among Brazilian older adults. ELSI-Brazil, 2015–2016.

| **Sociodemographic and lifestyle characteristics** | **Diabetes** | **Hypertension** | **High cholesterol** | **Excess body weight** | **Abdominal obesity** | **Cardiovascular risk** |
| --- | --- | --- | --- | --- | --- | --- |
| **Age group (years)** |  |  |  |  |  |  |
| 60–69 | 17.0 | 58.6 | 34.9 | 44.5 | 69.7 | 48.5 |
| 70–79 | 20.8 | 65.2 | 30.3 | 42.9 | 72.7 | 51.8 |
| ≥80 | 19.8 | 61.1 | 23.4 | 37.6 | 71.9 | 44.9 |
| **p-value*** | 0.082 | 0.012 | <0.001 | 0.045 | 0.153 | 0.135 |
| **Sex** |  |  |  |  |  |  |
| Female | 19.3 | 64.8 | 40.3 | 50.0 | 83.5 | 57.1 |
| Male | 17.5 | 56.4 | 23.1 | 35.7 | 56.6 | 40.1 |
| **p-value*** | 0.221 | <0.001 | <0.001 | <0.001 | <0.001 | <0.001 |
| **Self-reported race/skin color** |  |  |  |  |  |  |
| White | 19.6 | 59.7 | 30.3 | 46.1 | 72.9 | 48.8 |
| Other** | 17.4 | 61.6 | 34.3 | 41.3 | 68.8 | 49.5 |
| **p-value*** | 0.138 | 0.314 | 0.027 | 0.008 | 0.053 | 0.694 |
| **Education** |  |  |  |  |  |  |
| Up to incomplete primary education | 17.1 | 55.7 | 33.0 | 45.6 | 71.7 | 45.6 |
| Complete primary education or more | 19.0 | 62.8 | 32.0 | 42.3 | 70.5 | 50.4 |
| **p-value*** | 0.283 | 0.002 | 0.681 | 0.143 | 0.560 | 0.028 |
| Household income per capita |  |  |  |  |  |  |
| < R$ 821,66 | 17.6 | 62.1 | 31.9 | 40.6 | 67.6 | 37.8 |
| ≥ R$ 821,66 | 19.2 | 59.7 | 32.5 | 45.7 | 74.0 | 39.4 |
| **p-value*** | 0.324 | 0.157 | 0.788 | 0.006 | <0.001 | 0.426 |
| **Place of residence** |  |  |  |  |  |  |
| Urban | 19.8 | 60.9 | 33.0 | 45.2 | 72.5 | 50.7 |
| Rural | 11.4 | 60.5 | 28.0 | 33.6 | 62.3 | 40.7 |
| **p-value*** | <0.001 | 0.871 | 0.018 | <0.001 | <0.001 | <0.001 |
| **Low consumption of fruits and vegetables** |  |  |  |  |  |  |
| Yes | 18.4 | 60.7 | 32.1 | 42.6 | 70.5 | 48.8 |
| No | 20.9 | 61.0 | 38.8 | 56.6 | 74.5 | 56.6 |
| **p-value*** | 0.441 | 0.940 | 0.084 | 0.005 | 0.444 | 0.034 |
| **Smoking** |  |  |  |  |  |  |
| Yes | 14.2 | 49.7 | 24.8 | 25.1 | 49.0 | 29.4 |
| No | 19.2 | 62.7 | 33.4 | 46.3 | 74.4 | 52.3 |
| **p-value*** | 0.020 | <0.001 | <0.001 | <0.001 | <0.001 | <0.001 |
| **Alcohol consumption** |  |  |  |  |  |  |
| Yes | 15.5 | 54.2 | 26.9 | 39.3 | 65.8 | 41.9 |
| No | 19.5 | 63.1 | 34.0 | 44.7 | 72.5 | 51.5 |
| **p-value*** | 0.012 | <0.001 | 0.003 | 0.007 | <0.001 | <0.001 |
| **Polypharmacy** |  |  |  |  |  |  |
| Yes | 35.8 | 81.5 | 42.2 | 56.7 | 82.7 | 62.3 |
| No | 14.6 | 56.4 | 30.0 | 40.3 | 68.3 | 14.9 |
| **p-value*** | <0.001 | <0.001 | <0.001 | <0.001 | <0.001 | <0.001 |

Note: *Pearson’s chi-square test with Rao–Scott correction. **Individuals self-identifying as Black, Asian, Brown (mixed race), or Indigenous.
